# Extratropical Forcing Triggered the 2015 Madden–Julian Oscillation–El Niño Event

**DOI:** 10.1038/srep46692

**Published:** 2017-04-24

**Authors:** Chi-Cherng Hong, Huang-Hsiung Hsu, Wan-Ling Tseng, Ming-Ying Lee, Chun-Hoe Chow, Li-Chiang Jiang

**Affiliations:** 1 Department of Earth and Life, University of Taipei, Taipei, Taiwan; 2 Research Center for Environmental Changes, Academia Sinica, Taipei, Taiwan; 3 Central Weather Bureau, Taipei, Taiwan; 4 Department of Oceanography, National Sun Yat-Sen University, Kaoshiung, Taiwan.

## Abstract

In this paper, we report the triggering effect of extratropical perturbation on the onset of an atypical Madden–Julian Oscillation (MJO) and onset of the 2015–16 El Niño in March 2015. The MJO exhibited several unique characteristics: the effect of extratropical forcing, atypical genesis location and timing in the equatorial western Pacific, and the extremity of amplitudes in many aspects. The southward-penetrating northerly associated with the extratropical disturbances in the extratropical western North Pacific contributed to triggering the deep convection and westerly wind burst (WWB) and onset of the MJO over the anomalously warm tropical western Pacific in early March. The persisting strong WWB forced downwelling Kelvin wave-like oceanic perturbation that propagated eastward and led to the onset of the 2015–16 El Niño. The proposed novel extratropical forcing mechanism explaining the unique extratropics–MJO–El Niño association, based on both data diagnostics and numerical experiments, warrants further attention for a more detailed understanding of the onset of the MJO and its potential effect on El Niño.

An El Niño event had been expected to occur since spring 2014 because of the high ocean heat content in the equatorial western Pacific. The actual onset of the most recent El Niño, which is comparable to the 1982–83 and 1997–98 events, did not occur until May 2015 following the occurrence of a Madden–Julian Oscillation (MJO) of unprecedented amplitude in the tropical western Pacific in March. Studies have reported the triggering effect of the MJO on El Niño^1^,^2^,^3^,^4^,^5^,^6^,^7^,^8^,^9^,^10^ and the amplitude asymmetry between the El Niño and La Niña^11^. An MJO might exert a reverse effect (e.g., causing the abrupt termination of the 1997–98 El Niño)^12^. By contrast, the warm ocean surface associated with an El Niño event might enhance an MJO through a more rigorous atmosphere–ocean interaction. Marshall *et al*.^13^ recently explored the MJO–sea surface temperature (SST) coupling in March 2015 and suggested that the anomalously warm SST intensified the MJO to an unusually higher amplitude and long duration, and the MJO consequently enhanced the El Niño development.

In March 2015, a strong MJO occurred in the equatorial western Pacific, a characteristic that is distinctively different from previous MJO–El Niño events (e.g., the 1997–98 El Niño event) in which the MJO often originated in the Indian Ocean (IO) and propagated eastward into the equatorial western Pacific. We explored this unique feature and found that the March 2015 MJO might have developed because of forcing originating in the extratropical western North Pacific. Studies have reported the extratropical forcing of the MJO^14^,^15^,^16^,^17^,^18^,^19^, although it might not frequently occur and subsequently emerge in the composite of many events because of its stochastic nature^7^,^8^,^20^,^21^. A similar process seemed to occur in late February 2015. The present study explored this possibility and its possible connection to the onset of the 2015–16 El Niño event.

In this study, we hypothesized the following dynamical processes. The southward-penetrating northerly associated with the extratropical disturbances facilitated triggering the deep convection and westerly wind burst (WWB), and the onset of the MJO over the anomalously warm tropical western Pacific in early March. The persisting strong WWB forced downwelling Kelvin wave-like oceanic perturbation that propagated eastward and led to the onset of the 2015–16 El Niño. We conducted data diagnostics and numerical simulations to provide supporting evidence.

## Results

### Role of the extratropical–tropical interaction on the Madden–Julian Oscillation onset

**Figure 1:**
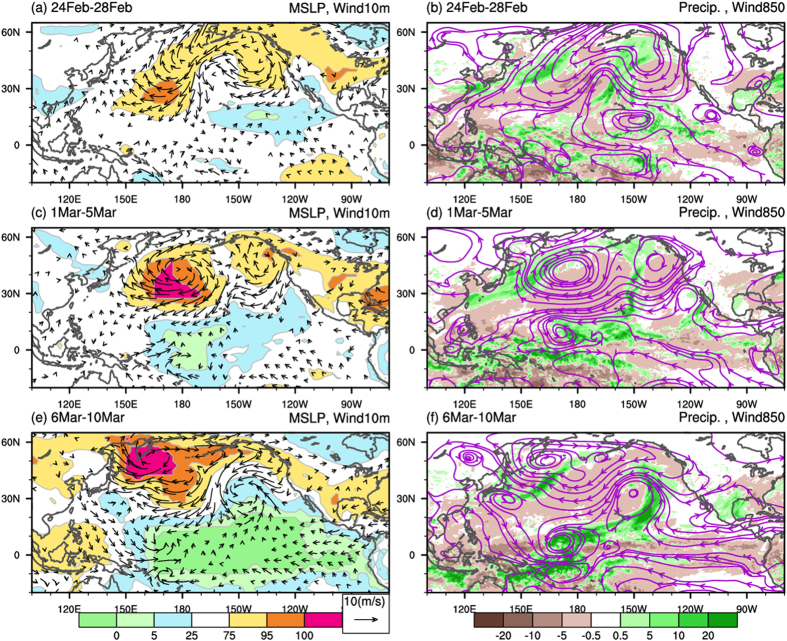
The 5-day means: (left panel) SLP (in percentile, shading), 2-m temperature (contour) and 10-m wind anomalies and (right panel) 850-hPa streamline and precipitation anomalies during (**a**,**b**) February 24–28, (**c**,**d**) March 1–5, and (**e**,**f**) March 6–10 in 2015. This map was created using NCAR command language software^35^.

**Figure 2:**
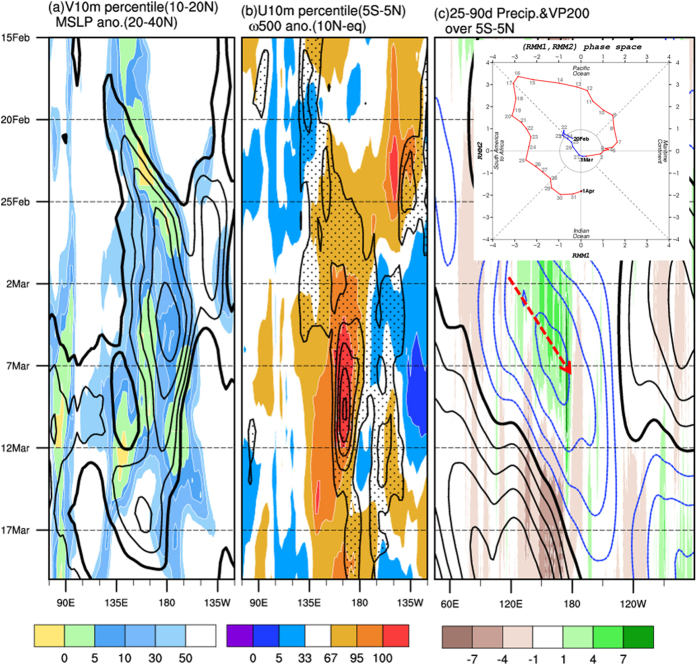
Hovmüller diagrams for February 15–March 17, 2015: (**a**) SLP (averaged over 20°N–40°N, contour) and 10-m meridional wind (averaged over 10°N–20°N, in percentile, shading) anomalies; (**b**) vertical velocity (in pressure coordinates, averaged over 0–10°N, contour) and 10-m zonal wind (averaged over 5°S–5°N, in percentile, shading) anomalies; (**c**) intraseasonal (25–90-day bandpass filtered) precipitation (shading) and 200-hPa velocity potential (averaged over 5°S–5°N). The inset in the upper corner of (**c**) denotes the onset of the MJO in the phase plane of the MJO index^24^. The arrow in (**c**) denotes the eastward propagation of the MJO. This map was created using NCAR command language software^35^.

Figure 1 presents the evolution of near-surface anomalous atmospheric fields during late February to early March in 2015. Before the MJO onset in early March, an atypically strong high-pressure system persisted over the extratropical western North Pacific in late February 2015. The accompanying dry and cold northerly to the west of the dateline penetrated southward to the tropical North Pacific, followed by the flaring of a deep convection in the equatorial western Pacific. The development of vertical circulation (averaged over 150°E–180°E; Supplementary Figure 1) further demonstrated the lead–lag relationship between the subsiding northerly in the extratropical western North Pacific and development of the deep convection in the tropical western Pacific. The Hovmüller diagrams shown in Fig. 2a further demonstrate the close temporospatial relationship between sea level pressure (SLP) and northerly anomalies in the subtropical North Pacific between 135°N and the dateline. The physical process of extratropical disturbance to trigger the MJO initiation was similar to the effect of the cold surge on the initiation of the intraseasonal oscillation^14^,^18^. The extratropical disturbance associated northeasterly anomaly advected cool and dry air southward to the warm and moist tropics. The cool and dry air above the warm moist ocean surface produced an unstable atmospheric environment favorable for the development of deep convection.

Following the arrival of extratropically originated perturbations, a tropical convection and strong WWB occurred to the west of the dateline in early March (Fig. 2b). As shown in Fig. 1e and f, the westerly anomaly in the tropical western Pacific, the easterly anomaly in the tropical eastern Pacific, the negative SLP anomaly over the tropical Pacific east of 150°E, and the deep convection west of the dateline formed a pattern resembling the theoretical Gill–Matsuno perturbation^22^,^23^. The emergence of this convection–circulation pattern signaled the onset of an MJO that later strengthened to an unprecedented amplitude (MJO real-time multivariate index^24^ of 4.62 on March 16, 2015 compared with the previous record of 4.02 on February 14, 1985; http://www.bom.gov.au/climate/mjo/) during its eastward propagation over the tropical Pacific in March (Fig. 2c)^13^.

In contrast to the canonical MJOs, which typically originate in the tropical IO, the MJO in spring 2015 initiated in the western Pacific. Our analysis revealed that only 3 out of the 75 large-amplitude MJOs (occurring in 1975, 2013, and 2015; approximately 4%) during 1974–2015 initiated in the western Pacific (i.e., phase 4 of the MJO index^24^, Supplementary Figure 2). Notably, only in 2015, the MJO preceded the onset of an El Niño event. No eastward-propagating tropical signals from the west but strong extratropical perturbations were observed preceding the 2015 MJO event; this lead–lag relationship implies the potential extratropical effect on triggering the MJO. Furthermore, the percentile values reported in Figs 1 and 2 show the unprecedented amplitudes of the extratropical SLP, northerly, WWB, tropical SLP anomaly crossing the central and eastern equatorial Pacific (Fig. 1c), and the MJO strength. This observation revealed the distinct characteristics associated with this MJO: the effect of extratropical forcing, atypical genesis location and timing, and the extremity of amplitudes in many aspects.

The warm ocean surface in the equatorial western Pacific might be another favorable condition conducive to MJO occurrence. A vertical cross section of the monthly ocean temperature along the equator (Supplementary Figure 3) revealed that the upper ocean in the central Pacific was approximately 1.5 K warmer in February–April 2015 than the long-term mean. This warming in the central Pacific was markedly higher than that during the onset of the 1997–98 El Niño. A previous study^13^ reported the effect of this warm water on MJO development in early spring 2015. An analysis of the SST evolution since early 2014 indicated that the warm SST in the central Pacific was primarily the remaining positive SST anomaly from the aborted 2014 El Niño (data not shown). The extratropical forcing in late February likely triggered the anomalous convection over this warm water and initiated the rigorous atmosphere–ocean interaction in the tropical western and central Pacific and onset of a strong MJO event.

### Effect of Madden–Julian Oscillation on triggering the 2015–16 El Niño onset

**Figure 3:**
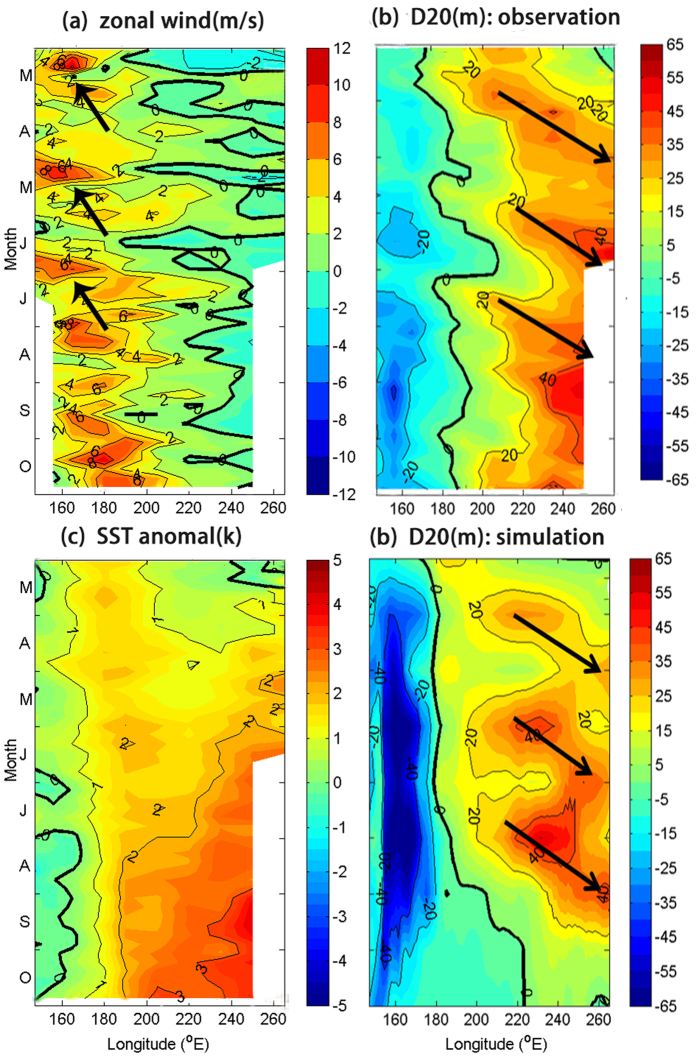
Hovmüller diagrams (averaged over 2°S–2°N) of (**a**) 10-m zonal wind anomalies, (**b**) 20 °C isotherm depth, and (**c**) SST anomalies in 2015 from TAO array. The arrows in (**a** and **b**) indicate the WWBs and eastward-propagating warm water mass, respectively. (**d**) Same as (**b**), except for the POP2 simulation. This map was created using NCAR command language software^35^.

Supplementary Figure 3 shows that the deep warm water mass started moving eastward in March in both the 2015 and 1997 El Niño events. Preceding the mature phase of both events, four–five pulses of the WWB occurred in the tropical western Pacific (Fig. 3a and Supplementary Figure 4) and triggered downwelling Kelvin wave-like ocean perturbations moving eastward and reaching the tropical eastern Pacific (Fig. 3b and Supplementary Figure 4) a couple of months later. Notably, the wind anomalies associated with the WWB in 2015 reached 10 m/s, approximately two standard deviations higher than the climatological mean. These results suggested that the first pulse of eastward-moving oceanic Kelvin wave-like perturbations, which arrived in the eastern Pacific in May, was triggered by the MJO-associated WWB. WWBs fluctuated at the intraseasonal time scale, but only the first burst was associated with an eastward-propagating MJO event. Studies^1^,^2^,^3^,^4^,^5^,^6^,^7^,^8^,^9^,^10^ have reported the effect of the MJO on triggering oceanic Kelvin wave-like perturbations. However, the forced oceanic Kelvin wave-like perturbations typically originated in the western Pacific (e.g., the first pulse in 1997, Supplementary Figure 4). Only a few cases, such as the 2015 event, initiated near the dateline (Fig. 3b). The WWB location was the critical factor, which was evident by comparing the 2015–16 and 1997–98 El Niño events (Fig. 3a and b and Supplementary Figure 4). Both El Niño events demonstrated a similar temporospatial evolution of the atmospheric and oceanic fields. The first oceanic Kevin wave-like perturbation in 1997 initiated in the western Pacific (150°E), whereas the 2015 event occurred near the dateline. This distinction resulted from the difference in the genesis location of the 1997 and 2015 MJO events: for example, the IO versus the west of the dateline (Supplementary Figure 5).

The main physical processes for an MJO triggering the El Niño onset are described as follows. The MJO-associated deep convection and strong low-level westerlies in the west cool the underlying SST through the cloud–radiation–SST and wind–evaporation–SST feedback. The subsidence branch of the anomalous west–east overturning circulation in the east enhances the downward shortwave radiation and warms the SST in the eastern Pacific. Therefore, the MJO-associated atmospheric circulation anomalies create a cool–warm SST contrast between the equatorial western and eastern Pacific, which provides favorable conditions for El Niño development. Furthermore, the MJO forces an eastward-moving downwelling Kelvin wave-like perturbation that directly contributes to the SST increase in the equatorial eastern Pacific. The aforementioned processes are clearly consistent with the observed features in 2015, suggesting the triggering effect of the MJO on the onset of the 2015–16 El Niño. The MJO, with a time scale markedly shorter than that of El Niño, is supposed to be a stochastic forcing in El Niño development. We investigated the El Niño events occurring after 1974. An El Niño onset was not necessarily preceded by an MJO, such as the 1982 event; 10 of 12 El Niño events were preceded by an apparent MJO signal (Supplementary Figure 2). This suggests that the MJO-related stochastic forcing is a major process in triggering El Niño onset.

### Numerical experiments

In this section, we provide evidence from two sets of numerical experiments (see Experimental Setting) to support our hypothesis. The first experiment is to address the effect of the MJO-associated WWB on forcing the oceanic Kelvin wave^6^,^7^,^8^. Figure 3d shows the Hovmüller diagrams (averaged over 2°S–2°N) of the simulated depth of 20 °C (D20) obtained from the oceanic simulations that were forced by three prescribed pulses of westerly wind stress. Three pulses of eastward warm water starting in March, May, and July were simulated (Fig. 3b). The simulated eastward propagation speed of warm water was approximately 1.7 ms^−1^, slightly slower but comparable with the observed propagation speed of approximately 2–3 ms^−1^. The numerical experiment confirmed that the observed oceanic Kelvin wave-like perturbations were triggered by the MJO-associated WWB. Moreover, the experiment revealed that the eastward-moving oceanic perturbation resulted in substantial SST warming in the central and eastern Pacific (data not shown).

**Figure 4:**
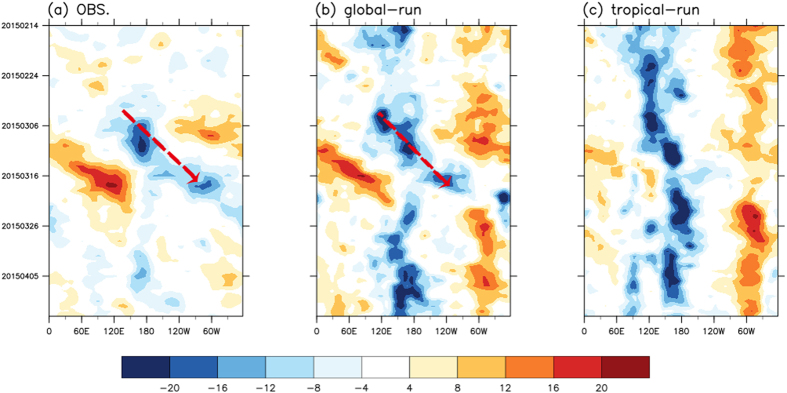
Hovmüller diagrams of 200-hPa velocity potential anomalies (averaged over 5°S–5°N; 10^−6^ 1/s): (**a**) NCEP-R1, (**b**) global-nudging simulation, and (**c**) tropical-nudging simulation. The arrows indicate the eastward propagation of the MJO. (**b** and **c**) present the compilations of the simulated velocity potential at Day 13 of 59 hindcast simulations starting from February 1 to March 31. Day 13 was selected for the visualization purpose only. The results are similar when Day 12 and Day 14 were chosen. This map was created using NCAR command language software^35^.

The second set of expriments examines the effect of extratropical forcing on initiating the MJO. The Hovmüller diagram of 200-hPa velocity potential of the atmospheric experiment shown in Fig. 4b and c is a compilation of the Day 13 simulated results from 59 global- and tropical-nudging simulations, respectively. The global-nudging simulations successfully reproduced the eastward-propagating signals (Fig. 4a), whereas the tropical-nudging simulations mainly produced a stationary convection to the west of the dateline. Plots for 10-m winds presented in Supplementary Figure 6 indicate the more realistic simulation of the WWB in the global-nudging simulations; this contrast was evident in individual simulations. Supplementary Figure 7 presents the results of two simulations starting from February 22 and 27 and provides a more direct view of the simulation results. The evident improvement in the global-nudging simulations compared with the tropical-nudging simulations supported our hypothesis that the extratropical perturbations contributed to triggering the MJO onset in early March 2015.

The extratropical forcing in triggering the MJO onset was primarily from the North Pacific. By contrast, no similar extratropical disturbances in the South Hemisphere were observed (Fig. 1a and Supplementary Figure 1). To further confirm this argument, a new experiment, the NP-nudging run, in which the nudging by the observed extratropical daily fluctuations was applied only to the North Pacific (10°N–90°N, 110°E–130°W) to isolate the origin of major extratropical forcing. The simulated 10-m zonal winds of the NP-nudging run resemble that of the global-nudging run, indicating that the extratropical forcing that triggered the westerly wind burst associated with the MJO was primarily from the North Pacific.

## Conclusion and Discussion

An atypical MJO initiated to the west of the dateline in early March 2015 and rapidly amplified to an unprecedented magnitude over the warm SST in the central and eastern Pacific on March 16. Following the MJO, the SST in the equatorial central–eastern Pacific encountered rapid growth and ultimately evolved to a strong El Niño comparable with the 1982–83 and 1997–98 events. Before the MJO onset, we observed a persisting high-pressure system accompanied by strong cold northerly in the extratropical western North Pacific. On the basis of data diagnostics and numerical experiments, we identified an atypical effect of extratropical perturbations in the western North Pacific on triggering the onset of the MJO in March 2015 and indirectly contributing to the onset of the 2015–16 El Niño. The main results are summarized as follows:

1. Observational analysis indicated that the strong cold northerly, which was associated with a persisting high-pressure system in the extratropical western North Pacific, penetrated southward to the tropical western Pacific and triggered the tropical convective instability that led to the onset of the MJO at an atypical location, namely west of the dateline. The critical effect of the extratropical disturbances on the MJO onset was confirmed by numerical experiments by using an atmospheric general circulation model coupled with an ocean mixed layer model.
2. The MJO developed rapidly to an extreme magnitude because of the favorable ocean conditions: a warm upper ocean temperature in the equatorial central and western Pacific remaining from the aborted 2014 El Niño.
3. Both data diagnostics and numerical experiments revealed that the strong WWB associated with the MJO triggered the first pulse of downwelling Kelvin wave-like perturbations that later induced the onset of the 2015–16 El Niño.

Extratropical forcing was the unique characteristic of the reported MJO–El Niño event. The onset of El Niño by an MJO has been observed often. However, according to our review of relevant literature, the present study is the first to report the onset of an El-Niño-inducing MJO in the western Pacific triggered by extratropical perturbations. Extremity was another unique characteristic. Several aspects of perturbations, such as extratropical and tropical SLP, northerly, and the MJO reached unprecedented amplitudes. The reasons for these unique characteristics remain unknown. However, our study revealed the possible effect of extratropical forcing, which has not been considered previously, on the onset of MJO and El Niño. Such a mechanism, although it might not occur frequently, warrants further attention and may elucidate the onset of an MJO and its potential effect on El Niño.

## Methods

### Data

The following observational data were used in this study: (1) the daily averaged atmospheric data of NCEP/NCAR Reanalysis I^25^, including SLP, 10-m winds, pressure-coordinate horizontal winds and vertical velocity, (2) NOAA/CPC Morphing Technique global precipitation^26^, (3) the SST from Met Office Hadley Center^27^, and (4) tropical atmosphere ocean (TAO) array data (available at http://www.pmel.noaa.gov/tao/proj_over/taohis.html), including 2-m zonal wind and D20 (depth of 20 °C isotherm). Percentile is presented to show the extremity of the anomalies. The percentile presented in Figs 1 and 2 is a measure used in the statistics indicating the value below which a given percentage observations in a group of observations fall. Here atmospheric variables from 1948–2015 were ranked from the smallest to the largest values. The 95 (5) percentile indicates the observation was in the top (lowest) five percent of observed values.

### Description of the atmospheric simulations

The atmosphere–ocean (mixed layer) coupled model ECHAM5-SIT, which realistically simulates MJO^28^,^29^, was used to investigate the triggering effect of extratropical forcing on the onset of an MJO. To identify the effect of extratropical perturbations, we conducted a series of hindcast experiments initialized at 00UTC daily from February 1 to March 31. The simulations were nudged toward the observed U, V, T and Q twice daily in the first 5 days and once daily on Days 6–10; thereafter, the nudging was stopped. In the global-nudging experiment, the simulations were nudged toward the observed daily global perturbations. In the tropical-nudging experiment, nudging by observed daily fluctuations was applied to the 10°S–10°N tropical band and by climatological means elsewhere. Because extratropical nudging was the main difference between two experiments, a more realistic simulation in the global-nudging experiment can be attributed to the forcing effect of the extratropical perturbations. A North Pacific (NP) nudging experiment was also conducted, by applying nudging to the North Pacific (10°N–90°N, 110°E–130°W) and the 10°S–10°N tropical band. An intercomparison among three experiments further reveals the origin of extratropical forcing in the North Pacific.

### Description of the oceanic simulations

#### Model Description

The ocean model Parallel Ocean Program version 2 (POP2) was used to investigate the triggering effect of the MJO on the onset of El Niño. We used the Community Earth System Model (CESM) version 1.0.4 released by the National Center for Atmospheric Research. The CESM is a fully coupled, global climate model that provides state-of-the-art computer simulations of past, present, and future climate states of the earth. The ocean component of the CESM is the POP2 of the Los Alamos National Laboratory^30^. It is a level-coordinate ocean general circulation model that solves three-dimensional primitive equations for ocean dynamics by using hydrostatic and Boussinesq approximations. It uses a displaced North Pole grid with a nominal 1° horizontal resolution; the meridional resolution is increased to 0.27° near the equator. Moreover, 60 vertical levels are present, monotonically increasing from 10 m in the upper ocean to 250 m in the deep ocean. Previous studies have reported additional details^31^,^32^.

#### Experimental Setting

In the present study, we conducted two simulations following the Coordinated Ocean–Ice Reference Experiment II protocol^32^,^33^, which uses interannual varying atmospheric forcing data sets for the 1948–2007 period. The initial conditions for two simulations were identical, starting from the March of the year 371, corresponding to the 60-year repeated forcing in March 1958. Without any changes, a control simulation (control case) was continuously integrated for 8 months until October of the same year. To evaluate the tropical ocean response under strong winds, a simulation (test case) was conducted by uniformly adding 10 m/s to the zonal winds within 140°E–180°E and 3°S–3°N in March, May, and July. We selected 10 m/s on the basis of the 8-m/s westerly wind anomaly at 850 hPa observed in 2015. The 10-m/s of the westerly anomaly is approximately 0.14 N/m^2^ of the zonal wind stress forcing to the ocean model^34^. For simplification, the uniform 10 m/s westerly anomaly was applied instead of using the observed wind fields, to simulate three pulses of westerly winds occurring from March to July in 2015 (Fig. 3a) which were assumed to trigger three oceanic Kelvin Waves at the Equator (Fig. 3b). Another experiment using the observed wind stress anomaly as forcing, which was more persistent than the three idealized westerly events, was also conducted. In this experiment, the separation of three oceanic events was not as clear as shown here but the first eastward-propagating Kelvin wave initiated in March was clearly simulated.

### Figure Source

All the figures were created by authors using NCAR Command Language (NCL)^35^ [version 6.3.0], a product of the Computational & Information Systems Laboratory at the National Center for Atmospheric Research (NCAR) and sponsored by the National Science Foundation, is a free interpreted language designed specifically for scientific data processing and visualization.

## Additional Information

**How to cite this article:** Hong, C.-C. *et al*. Extratropical Forcing Triggered the 2015 Madden–Julian Oscillation–El Niño Event. *Sci. Rep.* **7**, 46692; doi: 10.1038/srep46692 (2017).

**Publisher's note:** Springer Nature remains neutral with regard to jurisdictional claims in published maps and institutional affiliations.

## Supplementary Material

Supplementary Dataset 1
