# Supplementary material for: Extratropical Forcing Triggered the 2015 Madden–Julian Oscillation–El Niño Event
Source: Sci Rep. 2017 Apr 24;7:46692. doi: 10.1038/srep46692 (PMC5402264; doi:10.1038/srep46692)
Supplement: Supplementary Dataset 1 [file srep46692-s1.doc]

Extratropical Forcing Triggered the 2015 Madden–Julian Oscillation–El Niño Event

Chi-Cherng Hong^1^, Huang-Hsiung Hsu^2^, Wan-Ling Tseng^2^, Ming-Ying Lee^3^, Chun-Hoe Chow^2, 4^, and Li-Chiang Jiang^2^

^1^Department of Earth and Life, University of Taipei, Taipei, Taiwan

^2^Research Center for Environmental Changes, Academia Sinica, Taipei, Taiwan

^3^Central Weather Bureau, Taipei, Taiwan

^4^Department of Oceanography, National Sun Yat-Sen University

Corresponding author: Huang-Hsiung Hsu, Research Center for Environmental Changes, Academia Sinica, 128 Academia Road, Section 2, Nankang, Taipei, 115, Taiwan

Email: hhhsu@gate.sinica.edu.tw

**Supplementary Data**

**Supplementary Figure 1** Pressure–latitude cross section (averaged over 150°E–180°E) of (v and w) winds in the periods (a–c) pre-MJO onset (February 23–25, 2015) and (d–f) MJO onset (March 4–6, 2015). The shading indicates the vertical velocity value in pressure coordinates. This map was created using NCAR command language software^1^.


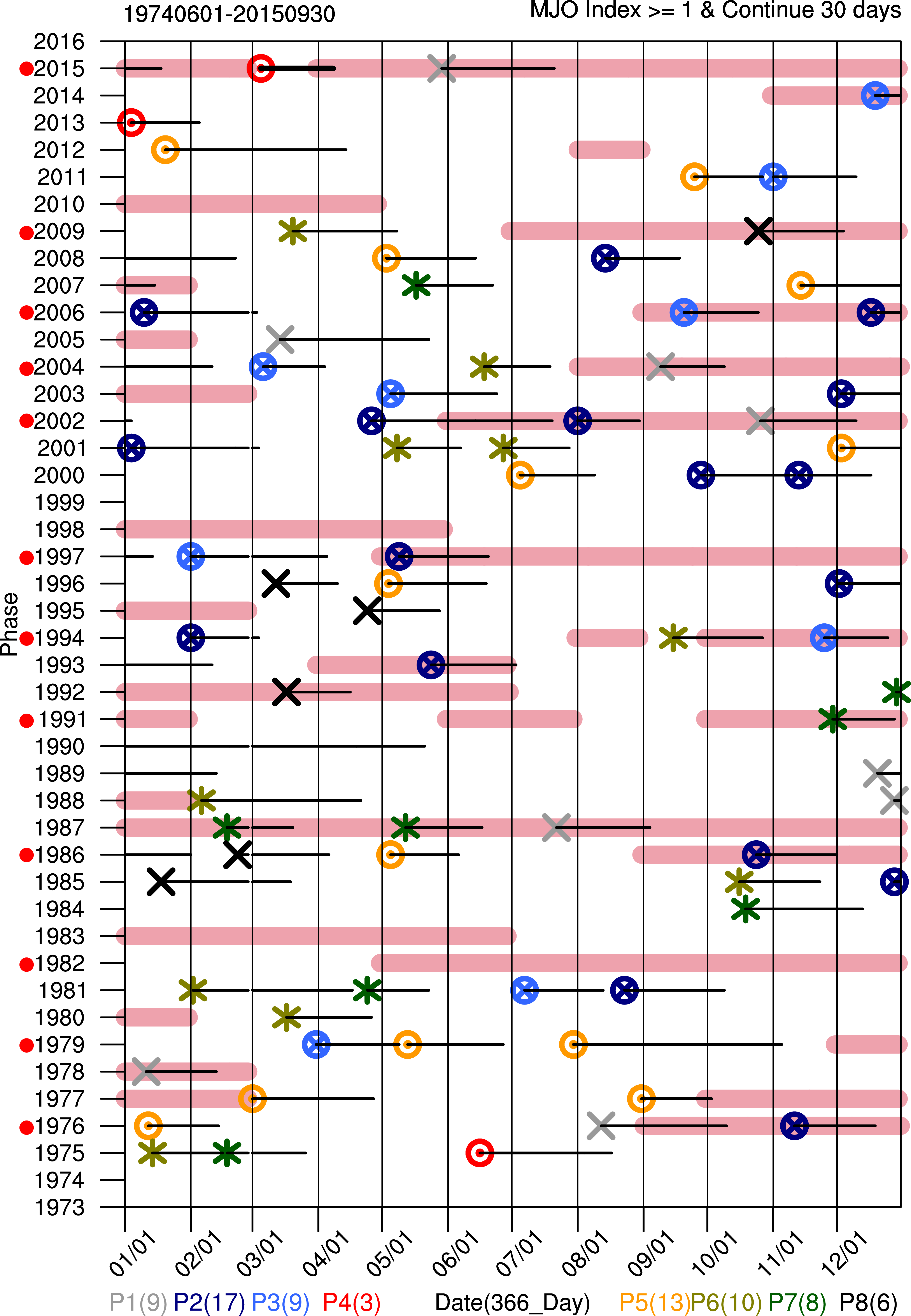


**Supplementary Figure 2** Statistics of strong and long-lasting MJOs during 1973–2016. The MJO, with a magnitude of the MJO index^19^ larger than 1 and sustaining for more than 30 days, was selected and shown. Seventy-five MJO events were selected and classified into eight groups (P1–P8, expressed using different markers) on the basis of the genesis location of the MJO. For example, P4(3) indicates the MJO initiated in the western Pacific, and the number in parentheses denotes the number of P4-type MJOs observed during 1973–2016. As indicated, only 3 of the 75 MJO events (occurring in 1975, 2013, 2015, approximately 4%) initiated in the P4 phase. The color of the circle indicates the type of MJO occurring at a certain time of a specific year, and the line trailing the circle denotes the period when the amplitude of an MJO remained larger than 1. The year marked with “●” indicates the onset year of an El Niño event. The red shaded bar indicates the 3-month running of Niño3.4 index is larger than 0.5 K. This map was created using NCAR command language software^1^.


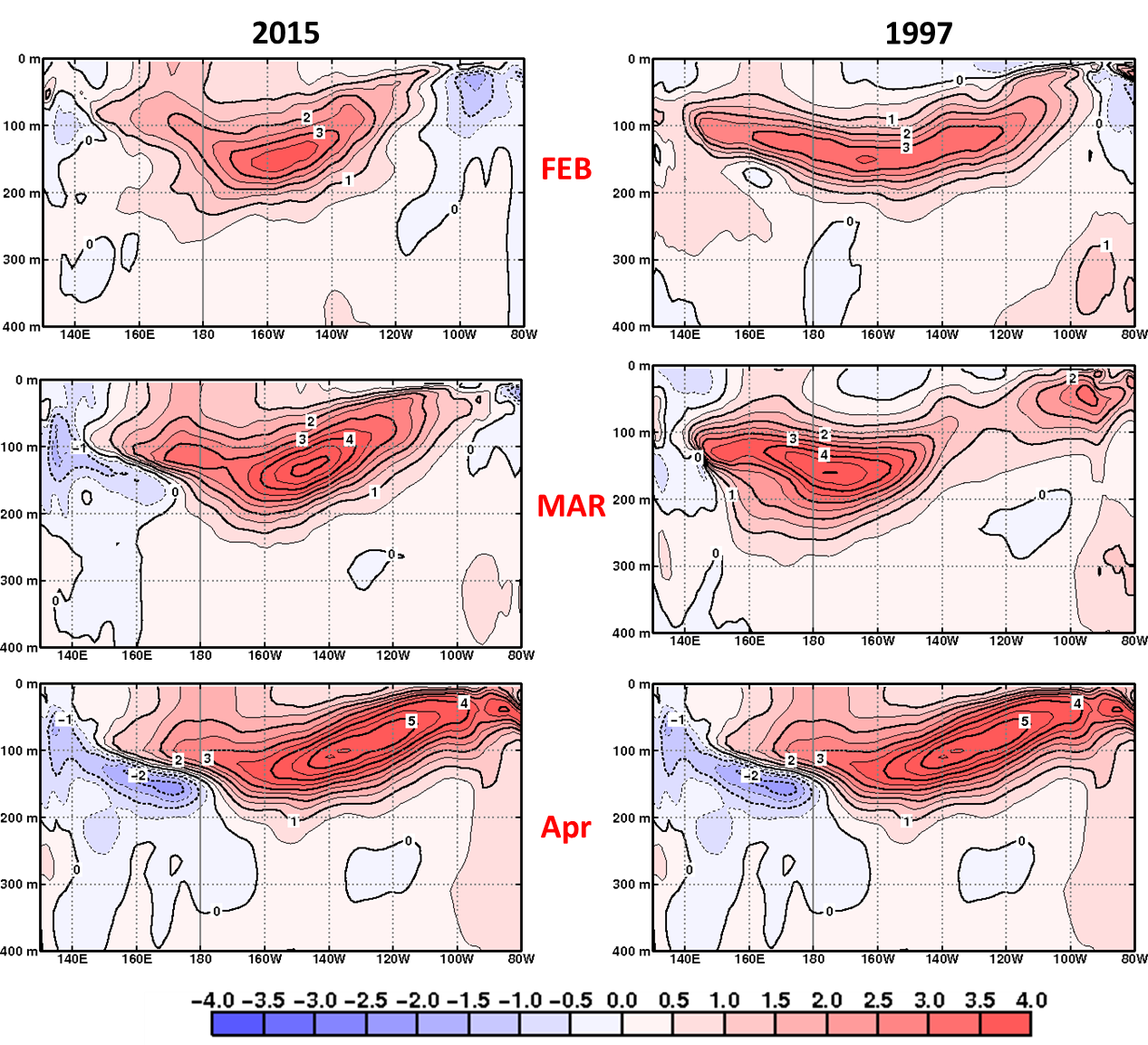


**Supplementary Figure 3** Vertical cross section (averaged over 2°S–2°N) of ocean temperature anomalies in February, March, and April 2015 (left panels) and 1997 (right panels). This map was created using NCAR command language software^1^.

**Supplementary Figure 4** Hovmüller diagrams (averaged over 2°S–2°N) of (a) the 10-m zonal wind, (b) 20 °C isotherm depth, and (c) SST anomalies in 1997. The arrows in (a) and (b) indicate the WWBs and eastward-propagating warm water mass. This map was created using NCAR command language software^1^.

**Supplementary Figure 5** Hovmüller diagram (averaged over 5°S–5°N) of the 10-m zonal wind anomalies in (a) 1997 and (b) 2015 from NCEP Reanalysis I. The arrows indicate the genesis location of MJO. This map was created using NCAR command language software^1^.

**Supplementary Figure 6** Same as Figure 4, except for the 10-m zonal wind (m/s). The red box indicates the period of the MJO onset. This map was created using NCAR command language software^1^.


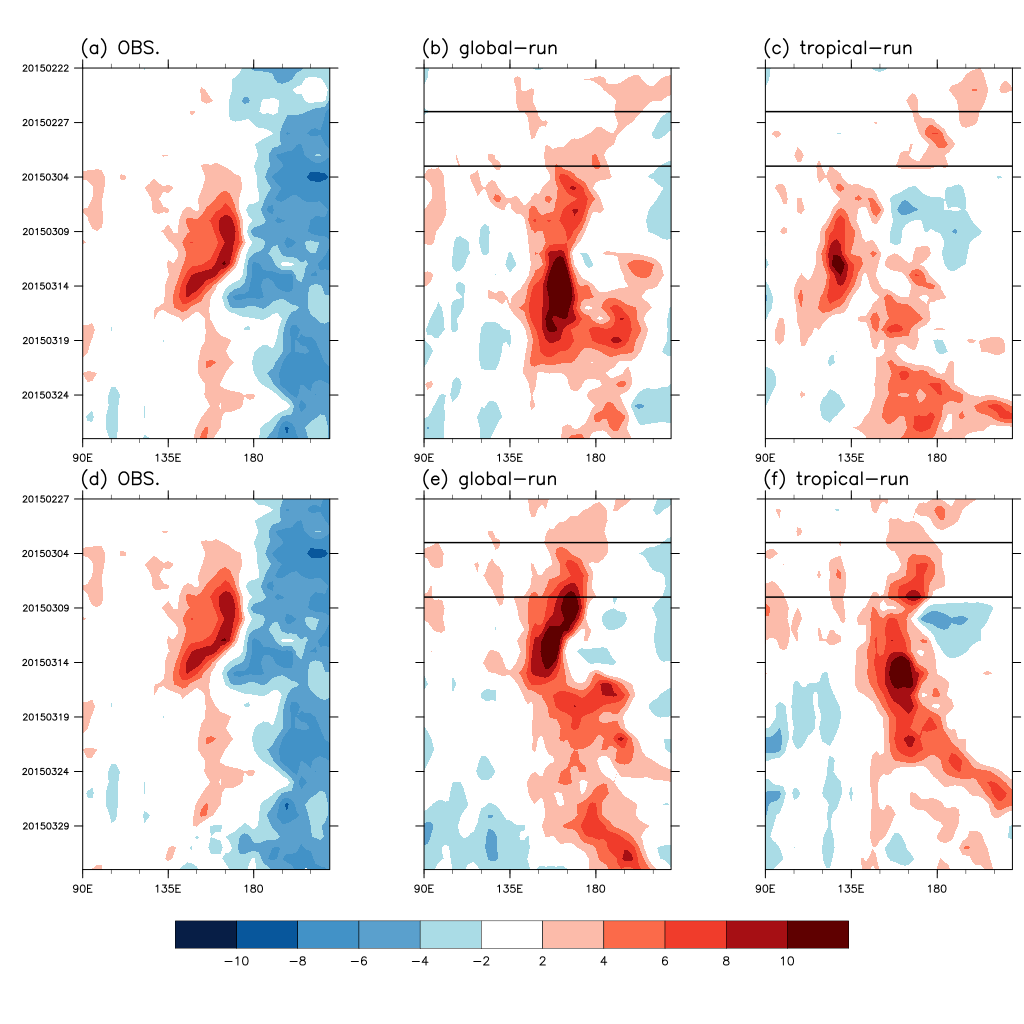


**Supplementary** **Figure 7** Observed (left) and simulated 10-m zonal wind initialized at 0000UTC on February 22 (top panel) and 27 (lower panel), 2015: global-nudging (middle) and tropical-nudging (right) simulations. The first and second horizontal lines indicate Days 5 and 10 of the simulations, respectively. This map was created using NCAR command language software^1^.

**References**

1. The NCAR Command Language (Version 6.3.0) [Software]. (2016). Boulder, Colorado: UCAR/NCAR/CISL/TDD.[http://dx.doi.org/10.5065/D6WD3XH5](https://mail.utaipei.edu.tw/owa/redir.aspx?C=1svU528Lakm-EeGKI4WPn8Jcb1aR_9MIzbesk15TFee-gmn2KmYP4PLCebWTADhEQfk5oOLdxNQ.&URL=http%3A%2F%2Fdx.doi.org%2F10.5065%2FD6WD3XH5)
